# Quinolone Safety and Efficacy

**DOI:** 10.3201/eid1106.040740

**Published:** 2005-06

**Authors:** Spartaco Bellomo

**Affiliations:** * Christ Hospital, Jersey City, New Jersey, USA

**Keywords:** efficacy, fluoroquinolones, safety

**To the Editor:** Richard Frothingham should be commended for providing added perspective on the matter of quinolone selection. His letter to the editor emphasizing the paramount importance of a well-established safety profile and documented clinical efficacy in severe infections before a "wholesale change" to the newer quinolones is an appropriate response to Michael Scheld's essay on maintaining quinolone class efficacy in which a "correct spectrum" strategy of using the most potent quinolone to treat the presumed or confirmed pathogen was described and advocated (1). In his article, Frothingham reminds us that serious adverse drug effects in patients led to the withdrawal or restriction of 4 quinolones in the last decade and that safety may differ substantially among the quinolones discussed in Scheld's review (ciprofloxacin, gatifloxacin, levofloxacin, moxifloxacin) (2).

With the exception of labeling changes regarding glucose homeostasis abnormalities associated with gatifloxacin therapy, the subject of quinolone safety is centered on torsades de pointes. Data published in 2001 are cited; these consist of a review of crude rates of US cases of torsades de pointes from January 1996 through May 2, 2001 (3). However, these data only capture adverse drug reports for the first full year gatifloxacin and moxifloxacin were widely available in the United States. The last several years have seen dramatic uptake of all 3 respiratory quinolones. Use of these agents is pervasive in both community and hospital settings. Indeed, the Infectious Diseases Society of America, American Thoracic Society, and Sinus and Allergy Health Partnership have since published revised consensus statements calling for the use of these agents earlier in therapy for community-acquired pneumonia and bacterial sinusitis (4–6).

December 2004 marked 5 years since the Food and Drug Administration approved gatifloxacin and moxifloxacin and 8 years since the approval of levofloxacin. As a result of tens of millions of patient exposures, we now have more robust data to work with and are better able to make informed and meaningful safety comparisons, particularly with respect to torsades de pointes, a rare, life-threatening cardiac arrhythmia infrequently associated with quinolone therapy.

With respect to efficacy, Frothingham writes that ciprofloxacin and levofloxacin have been studied in patient populations with more severe illness, and trials of the newer quinolones have enrolled patients with predominantly mild or moderate community-acquired infections and low overall death rates in comparison. However, a cursory review of the literature suggests otherwise. As with gatifloxacin and moxifloxacin, few peer-reviewed, published data support the use of levofloxacin in the treatment of severe, life-threatening infections at the currently approved doses of 500 mg or 750 mg.

Indeed, the 2 references cited raise serious concern about the suitability of levofloxacin at currently recommended doses for severe and life-threatening infections. In File et al. (7) levofloxacin was studied in only 16 patients classified as having severe community-acquired pneumonia; in Norrby et al. (8) a dose of levofloxacin 500 mg every 12 hours was studied in severe community-acquired pneumonia. At this time, other published studies support the use of levofloxacin at a dose of 500 mg every 12 hours in severe and life-threatening infections: an approved regimen in Europe but not yet approved in the United States (9,10).

In summary, differences in quinolone safety are evidenced by labeling changes to gatifloxacin, the only quinolone to carry a specific warning regarding glucose homeostasis abnormalities. However, the incidence of torsades de pointes associated with each of these agents is ripe for further investigation as we pass the 5-year mark of approval for the new respiratory quinolones. An update of those data on the rate of torsades cited by Frothingham and published in 2001 would provide meaningful guidance to clinicians. Currently, with the exception of ciprofloxacin, each of these quinolones contains labeling guidance in the form of a warning (gatifloxacin, moxifloxacin) or a precaution (levofloxacin), and concurrent use with class IA (e.g., quinidine, procainamide) or class III (e.g., amiodarone, sotalol) antiarrhythmics should be avoided to reduce the risk of torsades de pointes per current product labeling.

Ciprofloxacin remains the only quinolone to date based on multiple, head-to-head, well-controlled, published trials to have established efficacy and safety in a severely ill patient population at approved doses. A paucity of published clinical data exist on the use of gatifloxacin, levofloxacin and moxifloxacin in hospitalized patients with severe, life-threatening infections. Therefore, the respective manufacturers must establish safety and efficacy in well-controlled studies with the resultant data made available in peer-reviewed journals before these agents are fully embraced for these infections.
