# Supplementary material for: Quinolone Safety and Efficacy
Source: Emerg Infect Dis. 2005 Jun;11(6):985–7. doi: 10.3201/eid1106.040740 (PMC3367587; doi:10.3201/eid1106.040740)
Supplement: Related texts

# Reply: Quinolone Safety and Efficacy

**DOI:** 10.3201/eid1106.050581

**Authors:** Richard Frothingham

**Affiliations:** * Veterans Affairs Medical Center and Duke University Medical Center, Durham, North Carolina, USA

**In response:** I agree. Expanding clinical experience makes a reappraisal of quinolone safety and efficacy timely (1). Through December 2004, >160,000,000 ciprofloxacin, 69,000,000 levofloxacin, 12,000,000 gatifloxacin, and 11,000,000 moxifloxacin prescriptions were filled at US retail pharmacies.

Dr. Bellomo requests an update of my previous report on torsades de pointes adverse drug events (ADEs) (2). I reviewed 16,868 U.S. Food and Drug Administration ADE reports associated with these 4 quinolones from November 1997 to September 2003 (3). My review identified the following numbers of unique US torsades de pointes ADEs: 3 ciprofloxacin, 51 levofloxacin, 37 gatifloxacin, and 20 moxifloxacin. Ciprofloxacin or nonquinolone antimicrobial drugs should generally be selected for patients with risk factors for QT interval prolongation (4-7).

Numerous published trials and extensive clinical experience support the safety and efficacy of ciprofloxacin. Generic oral ciprofloxacin is inexpensive. These factors make ciprofloxacin the quinolone of choice for nonrespiratory infections.

Gatifloxacin is associated with hypoglycemia and hyperglycemia ADEs, including death, at rates greatly exceeding those of other quinolones (3). A causal relationship between gatifloxacin and these ADEs is supported by animal data (8), manufacturer safety cohort studies (5), in vitro assays (9), a large randomized trial (10), and >20 published case reports. Gatifloxacin has no meaningful cost or efficacy advantage to balance this excess risk.

Clinical experience continues to support the safety of levofloxacin and moxifloxacin. Apart from torsades de pointes, my review did not identify specific safety concerns. Both drugs are effective for community-acquired respiratory infections, although clinical experience and published data are more extensive of levofloxacin than moxifloxacin. Levofloxacin has received FDA approval for nosocomial pneumonia (6). Dr. Bellomo notes that levofloxacin trials have used a variety of dosages; the optimal dosage for serious infections is unknown. Moxifloxacin has greater activity against *Streptococcus pneumoniae*, which could possibly prevent the emergence of resistance or lead to faster clinical responses in pneumococcal infections. Both moxifloxacin and levofloxacin are appropriate choices for community-acquired respiratory infections.

## References

[1] Bellomo S. Quinolone safety and efficacy [letter]. Emerg Infect Dis. 2005;11:985–6.

[2] Frothingham R. Rates of torsades de pointes associated with ciprofloxacin, ofloxacin, levofloxacin, gatifloxacin, and moxifloxacin. Pharmacotherapy. 2001;21:1468–72. 10.1592/phco.21.20.1468.3448211765299

[3] Frothingham R. Gatifloxacin associated with a 56-fold higher rate of glucose homeostasis abnormalities than comparator quinolones in the FDA spontaneous reporting database [abstract A-1092]. In: Abstracts of the 44th Annual Interscience Conference on Antimicrobial Agents and Chemotherapy; 2004 Oct 30–Nov 2; Washington (DC). Washington, DC: American Society for Microbiology; 2004. p. 19.

[4] Bayer Corporation. Cipro IV (ciprofloxacin) prescribing information. April, 2004. West Haven, CT.

[5] Bristol-Myers Squibb Company. Tequin (gatifloxacin) prescribing information. 2004 Nov. Princeton, NJ.

[6] Ortho-McNeil Pharmaceutical. Levaquin (levofloxacin) prescribing information. 2004 Oct. Raritan, NJ.

[7] Bayer Corporation. Avelox (moxifloxacin) prescribing information. 2004 Oct. West Haven, CT.

[8] Food and Drug Administration Center for Drug Evaluation. Medical officer review of NDA 21-061 and 21-062: gatifloxacin (Tequin). 1999 Dec [cited 2004 Nov 14]. Available from http://www.fda.gov/cder/foi/nda/99/21061_Tequin.htm

[9] Saraya A, Yokokura M, Gonoi T, Seino S. Effects of fluoroquinolones on insulin secretion and beta-cell ATP-sensitive K+ channels. Eur J Pharmacol. 2004;497:111–7. 10.1016/j.ejphar.2004.06.03215321742

[10] Cannon CP, Braunwald E, McCabe CH, Grayston JT, Muhlestein B, Giugliano RP, Antibiotic treatment of *Chlamydia pneumoniae* after acute coronary syndrome. N Engl J Med. 2005;352:1646–54. 10.1056/NEJMoa04352815843667
